# Supplementary material for: Adeno-associated viruses (AAVs) induce dose-dependent neonatal ventriculomegaly following intracerebroventricular administration
Source: bioRxiv. 2025 Dec 31:2025.12.30.697113. Preprint. [Version 1] doi: 10.64898/2025.12.30.697113 (PMC12776412; doi:10.64898/2025.12.30.697113)
Supplement: Supplement 1 [file media-1.pdf]

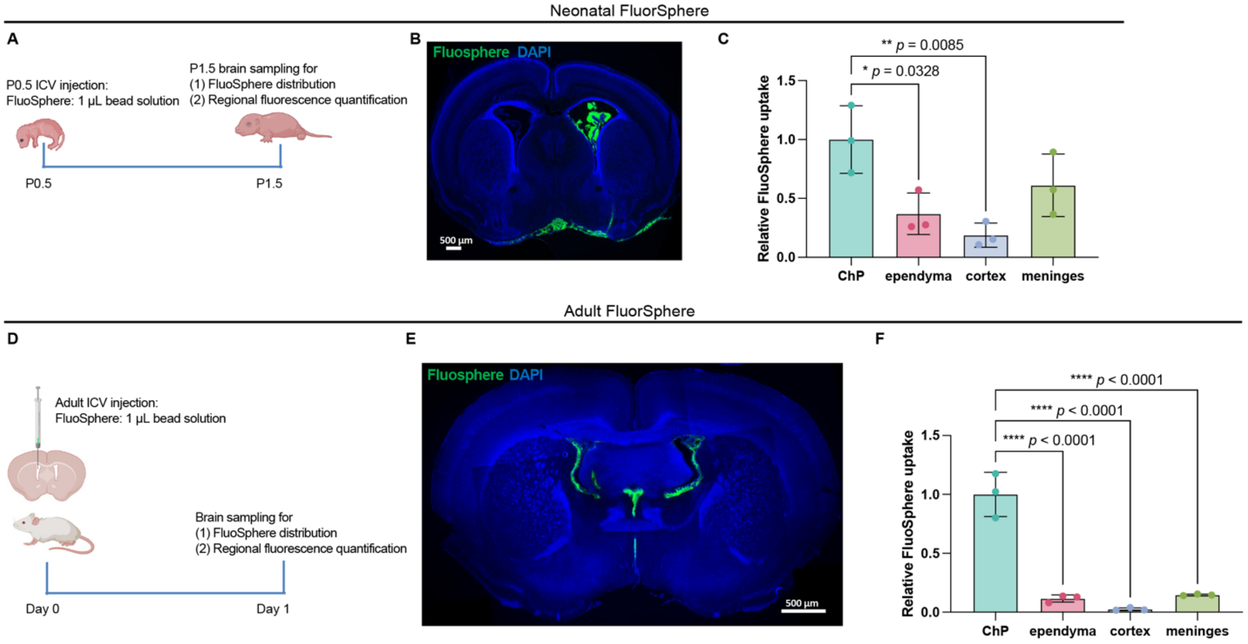

Supplementary Figure 1. Cellular uptake of AAV size- and charge-matched particles following ICV administration: the choroid plexus as the predominant site.

(A) Experimental timeline for ICV injection of FluoSphere beads in neonatal (P0.5) pups.

(B) Representative image showing the distribution of FluoSpheres (green) 24 hours post-ICV injection in a P1.5 neonatal brain.

(C) Quantitative analysis of FluoSphere fluorescence intensity across brain regions 24 hours post-injection in neonates (n = 3).

(D) Experimental timeline for ICV injection of FluoSphere beads in adult mice.

(E) Representative image showing FluoSphere distribution (green) 24 hours post-ICV injection in adult mouse brain.

(F) Quantitative analysis of FluoSphere fluorescence intensity across brain regions in adult mouse brain 24 hours post-injection (n = 3).

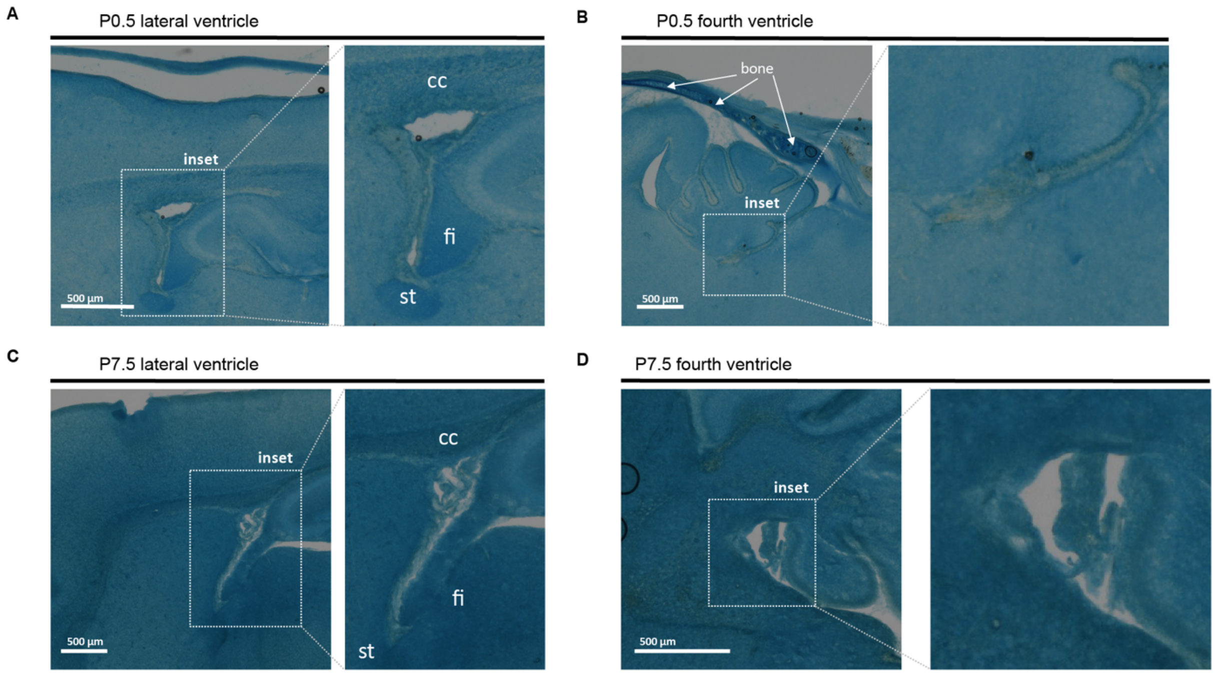

Supplementary Figure 2. Neonatal development of brain extracellular matrix anionic glycan molecules by Alcian blue staining.

(A) Alcian blue staining of P0.5 sagittal brain sections for the lateral and fourth ventricles. Insets highlight the characteristic periventricular staining pattern. *st*: stria terminalis; *fi*: fimbria; *cc*: corpus callosum.

(B) Alcian blue staining of P7.5 sagittal brain sections, showing increased staining intensity in periventricular regions. *st*: stria terminalis; *fi*: fimbria; *cc*: corpus callosum.

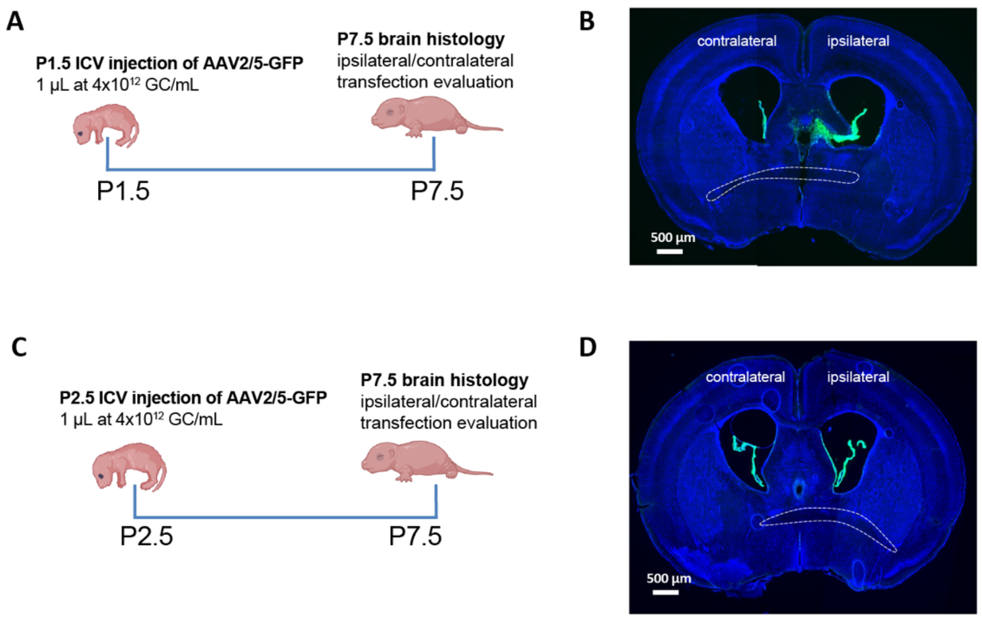

Supplementary Figure 3. Neonatal ICV injection timing influences the ipsilateral/contralateral transfection pattern, but not the ventriculomegaly outcomes.

(A) Experimental timeline for P1.5 ICV injection of high-dose AAV2/5 and analysis at P7.5.

(B) AAV2/5 transfection pattern (GFP) in P7.5 brains. The dashed line marks posterior anterior commissure (pAC).

(C) Experimental timeline for P2.5 ICV injection of high-dose AAV2/5 and analysis at P7.5.

(D) AAV2/5 transfection pattern (GFP) in P7.5 brains. The dashed line marks the pAC.

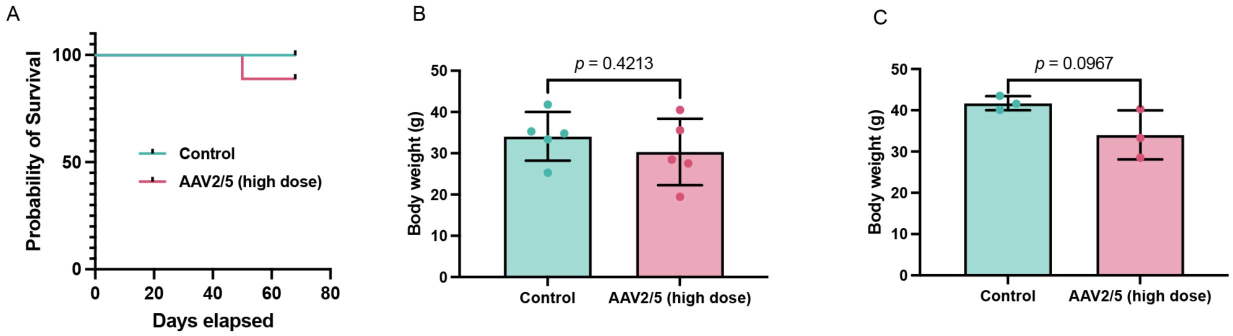

Supplementary Figure 4. Long-term health assessment following neonatal ICV injection.

(A) Survival curve tracked to postnatal day 68 following P0.5 ICV injections of high-dose AAV2/5 (n = 7-8 per condition).

(B, C) Body weight of female (n = 5 per condition) **(B)** and male (n = 3 per condition) **(C)** mice measured 8 weeks post-P0.5 ICV injection.

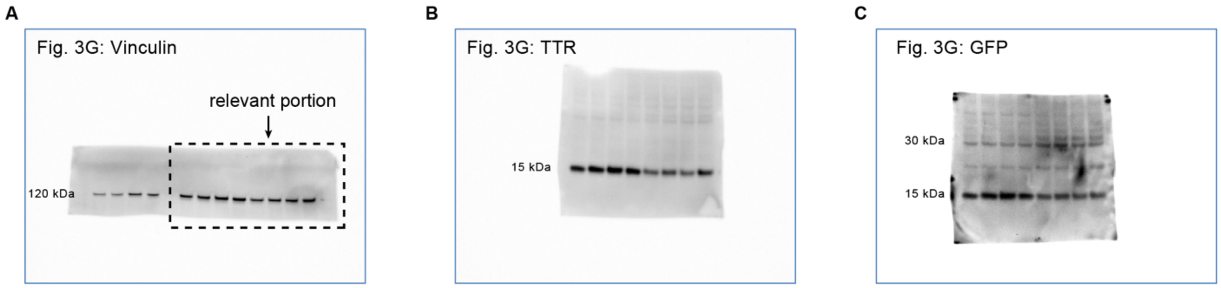

Supplementary Figure 5. Full western blot membranes corresponding to Figure 3G.

(A) Vinculin immunoblot.

(B) Transthyretin (TTR) immunoblot.

(C) The membrane from (B) was stripped and re-probed for GFP.
